# The unusual flagellar-targeting mechanism and functions of the trypanosome ortholog of the ciliary GTPase Arl13b

**DOI:** 10.1242/jcs.219071

**Published:** 2018-09-05

**Authors:** Yiliu Zhang, Yameng Huang, Amrita Srivathsan, Teck Kwang Lim, Qingsong Lin, Cynthia Y. He

**Affiliations:** Department of Biological Sciences, Faculty of Science, National University of Singapore, 14 Science Drive 4, Singapore 117543

**Keywords:** Arl13b, Arl3, Dimerization/Docking domain, Flagellum, *Trypanosoma brucei*

## Abstract

The small GTPase Arl13b is one of the most conserved and ancient ciliary proteins. In human and animals, Arl13b is primarily associated with the ciliary membrane, where it acts as a guanine-nucleotide-exchange factor (GEF) for Arl3 and is implicated in a variety of ciliary and cellular functions. We have identified and characterized *Trypanosoma brucei* (Tb)Arl13, the sole Arl13b homolog in this evolutionarily divergent, protozoan parasite*.* TbArl13 has conserved flagellar functions and exhibits catalytic activity towards two different TbArl3 homologs. However, TbArl13 is distinctly associated with the axoneme through a dimerization/docking (D/D) domain. Replacing the D/D domain with a sequence encoding a flagellar membrane protein created a viable alternative to the wild-type TbArl13 in our RNA interference (RNAi)-based rescue assay. Therefore, flagellar enrichment is crucial for TbArl13, but mechanisms to achieve this could be flexible. Our findings thus extend the understanding of the roles of Arl13b and Arl13b–Arl3 pathway in a divergent flagellate of medical importance.

This article has an associated First Person interview with the first author of the paper.

## Abstract (precis)

**Highlighted Article:** All roads lead to cilia – how the essential flagellar enrichment of Arl13 is achieved in trypanosome cells using a fundamentally different strategy compared with that of animal cells.

## INTRODUCTION

Flagella/cilia provide critical motility and sensory functions in eukaryotic cells from protists to mammals alike. Their roles in human physiology and diseases (ciliopathies) have spurred renewed interest in this ancient organelle (Badano et al., 2006; Hildebrandt et al., 2011). The cilium is topologically an open organelle (not fully enclosed by membrane), and relies on elaborate protein trafficking/gating systems to maintain its molecular identity and functions (Garcia-Gonzalo and Reiter, 2012; Ishikawa and Marshall, 2011). The small GTPase Arl13b localizes predominantly to the ciliary membrane in all ciliated model systems studied thus far and has been widely adopted as a ciliary marker (Cantagrel et al., 2008; Cevik et al., 2010). Studies have shown that Arl13b targeting involves multiple mechanisms, requiring at least two sequence elements essential for localization: an N-terminal palmitoylation motif that facilitates membrane association, and a less-conserved VxP motif-containing C-terminal tail that restricts the protein to the ciliary membrane. However, these elements are not exhaustive and other factors might also be at play (Cevik et al., 2013; Higginbotham et al., 2012; Hori et al., 2008). The ciliary localization of Arl13b is believed to be critical for its functions, as Arl13b transgenes that contain targeting-related mutations fail to rescue Arl13b loss-of-function phenotypes (Duldulao et al., 2009; Higginbotham et al., 2012).

Mutations in Arl13b are associated with Joubert syndrome (a ciliopathy), and Arl13b-null animals display a wide range of ciliary defects that compromise ciliogenesis, cilia length extension, ciliary motility and sonic hedgehog signaling (Caspary et al., 2007; Cevik et al., 2010; Duldulao et al., 2009; Larkins et al., 2011; Li et al., 2010; Lu et al., 2015; Sun et al., 2004). Concomitant with these pleiotropic phenotypes, various molecular mechanisms have been ascribed to Arl13b, including the regulation of intraflagellar transport (IFT) (Cevik et al., 2010; Li et al., 2010; Nozaki et al., 2017), cooperation with the exocyst (Seixas et al., 2015) and direct recruitment of other important ciliary proteins, such as INPP5E (Humbert et al., 2012). The best-studied and most-conserved role of Arl13b discovered so far is being a guanine-nucleotide-exchange factor (GEF) for Arl3 – another Arf/Arl family member implicated in ciliary functions (Cuvillier et al., 2000; Gotthardt et al., 2015; Hanke-Gogokhia et al., 2016; Ivanova et al., 2017; Schrick et al., 2006; Zhang et al., 2016). It has been proposed that the exclusive ciliary localization of Arl13b (Arl3-GEF) creates an Arl3-GTP gradient between the cilia and the cytoplasm, which in turn facilitates the directional transport of ciliary proteins. However, this model only applies to lipidated ciliary proteins, which may not readily explain the wide range of ciliary defects seen in Arl13b and Arl3 mutants.

Arl13b is believed to be one of the most ancient ciliary proteins (Sung and Leroux, 2013), yet little is known regarding its role outside of metazoan systems. We serendipitously discovered that in the uni-flagellated protozoan parasite *Trypanosoma brucei*, the single homolog of Arl13b (TbArl13) lacks both the palmitoylation motif and the C-terminus tail – both of which are essential for Arl13b ciliary targeting in higher eukaryotes. In light of recent evidence that Arl13b may also have roles outside of the cilia (Casalou et al., 2014; Mariani et al., 2016), we originally hypothesized that TbArl13 might represent an evolutionarily divergent, non-ciliary form of Arl13b. However, we found that TbArl13 is targeted to the flagellum by associating with the axoneme, through a domain similar to the dimerization/docking (D/D) domain of the protein kinase A (PKA) regulatory subunit in mammalian cells. Replacing the D/D domain in TbArl13 with the full-length protein sequence for calflagin Tb24, a trypanosome flagellar membrane-associated protein, enriches the chimera protein at the flagellar membrane. Interestingly, this chimeric TbArl13 mutant could functionally rescue cell death phenotypes upon depletion of endogenous TbArl13, suggesting that the ‘end’ of flagellar enrichment is more critical than the ‘means’ of targeting. Finally, we showed that the Arl13b–Arl3 pathway in *T. brucei* is conserved but bifurcated, in that TbArl13 catalyzes two differentially localized, functionally important Arl3 homologs.

## RESULTS

### TbArl13 is an Arl13b ortholog with unusual features

Reciprocal BLAST analyses have identified Tb927.10.5230 as a homolog of human Arl13b. Both E-values and identity/similarity scores are within the range reported for other published Arl13b homologs (Cevik et al., 2010; Fig. S1A). To further analyze Tb927.10.5230, we performed phylogenetic reconstruction, sampling all predicted trypanosome Arf/Arl GTPases and major Arf/Arl family members from representative eukaryotic model systems (Fig. S1B). The resulting phylogenetic tree confidently placed Tb927.10.5230 in the same clade with all other known Arl13/Arl13a/Arl13b proteins included in the dataset (Fig. S1B). Because the duplication of Arl13 to Arl13a and Arl13b is likely a vertebrate invention (Kahn et al., 2014), we denote the trypanosome ortholog as TbArl13 to reflect its homology to both Arl13a and Arl13b. Owing the scarcity of functional information on Arl13a, we limit our discussion to be in the context of the role of Arl13b in this work.

**Fig. 1.**
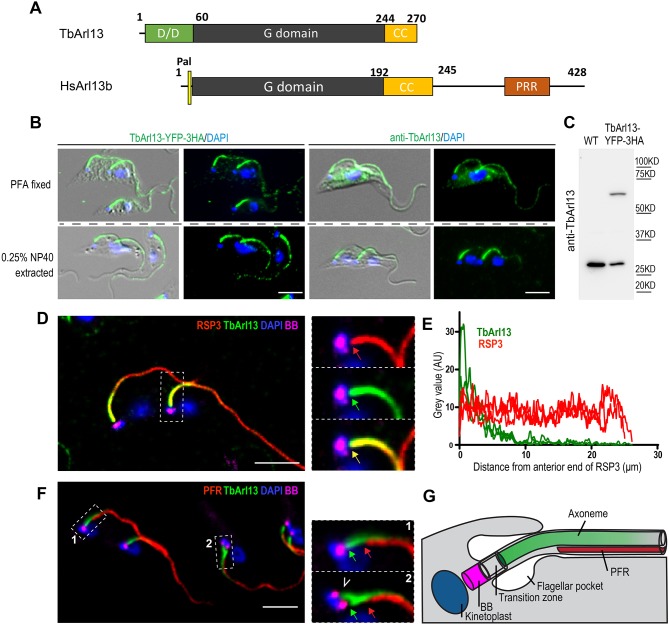
**TbArl13 associates with the flagellar axoneme.** (A) Domain organization of TbArl13 and human Arl13b. (B) Endogenously expressed TbArl13–YFP–3HA (labeled with anti-HA antibody; left) and native TbArl13 (labeled with anti-TbArl13 antibody; right) are both present in 0.25% NP40-extracted flagella. The cells are counter-stained with DAPI (blue) to visualize the DNA-containing nucleus (large oval) and kinetoplast (small oval near the base of each flagellum). Cells in different cell cycle stages, with single or duplicated organelles are shown. (C) The polyclonal anti-TbArl13 antibodies specifically recognize native TbArl13 and TbArl13–YFP–3HA on immunoblots. (D) Cells stably expressing YFP–RSP3 (radial spoke protein 3, Tb927.11.1150; red) in the axoneme were extracted with 0.25% NP40, fixed and labeled with anti-TbArl13 antibody (green), YL1/2 antibody for the basal bodies (BB; magenta) and DAPI (blue). Enlarged views of the proximal region of one flagellum (demarcated by dashed line) are shown on the right. Note that both TbArl13 (green arrow) and YFP–RSP3 (red arrow) signals initiate at the same site. (E) Representative plot profiles of fluorescence intensities of both YFP–RSP3 (red) and anti-TbArl13 antibody (green) along the axoneme, demonstrating the gradient distribution of TbArl13 along the axoneme. (F) An NP40-extracted cytoskeleton was co-stained with anti-PAR antibody to mark the paraflagellar rod (PFR; red), YL1/2 antibody (BB, magenta) and anti-TbArl13 antibody (green). Enlarged views of the proximal ends of a new flagellum (box 1) and a nascent flagellum formed just posterior to an existing flagellum (box 2) are shown on the right. The anti-TbArl13 antibody signals initiate at a position (green arrow) significantly closer to the BB than that of PFR (red arrow). Anti-TbArl13 antibody also stains the nascent flagellum (arrowhead), where no PFR is yet assembled. (G) Schematic drawing of the trypanosome flagellum, highlighting the relative position of the PFR and axoneme to the basal body. Scale bars: 5 μm.

Multiple sequence alignment and domain comparison of Arl13b proteins (Fig. 1A; Fig. S2) show that TbArl13 possesses a highly conserved GTPase domain, with characteristic absence of the ‘Switch 2’ catalytic glutamine residue (DxxGQ) that is highly conserved in other small GTPases (Cevik et al., 2010; Miertzschke et al., 2014). Immediately following the GTPase domain is a predicted coil-coil motif (CC), which is also unique to Arl13b proteins and required for their catalytic function (Hori et al., 2008; Miertzschke et al., 2014). Notably, TbArl13 does not have the N-terminal palmitoylation site found in most other Arl13b members except for *Chlamydomonas reinhardtii* (Cr)Arl13b (Fig. S2). TbArl13 also lacks a long C-terminal tail – another characteristic, although less-conserved feature of Arl13b (Li et al., 2010).

The most unusual feature of TbArl13 is the presence of an N-terminal extension, which is predicted to be a ‘dimerization/docking domain of the PKA regulatory subunit’ (D/D, NCBI conserved domain ID: cl02594; Superfamily ID SSF47391; Interpro ID: ipr003117). D/D is a class of protein targeting domains best characterized in PKA, but they have also been found in non-PKA proteins (Banky et al., 1998; Carr et al., 2001; Sivadas et al., 2012). The D/D domain is not present in Arl13b proteins of higher eukaryotes but is highly conserved among Arl13b homologs in the kinetoplastid group of protozoans (Fig. S3). Taken together, our bioinformatic analyses show that TbArl13 has highly conserved catalytic domains (a GTPase domain and the CC motif) but is divergent in elements that have been implicated in targeting and/or regulatory functions.

### TbArl13 localizes to the flagellum and associates with the axoneme

We first investigated the intracellular localization of TbArl13 in procyclic form (PCF) *T. brucei* cells (the proliferative form found in tse-tse fly midgut) by tagging one endogenous TbArl13 allele with a yellow fluorescent protein (YFP) reporter followed with a triple HA tag (Fig. 1B). Highly specific polyclonal antibodies were also raised against TbArl13 (Fig. 1C). In all three approaches, TbArl13 displayed flagellar localization with stronger signal intensity towards the proximal end (Fig. 1B–G). Some weak cytoplasmic signals were also observed in PFA-fixed whole cells (Fig. 1B, top row). The TbArl13 association with the flagellum was at least partially resistant to detergent extraction (Fig. 1B, bottom row), suggesting association with the flagellar cytoskeleton. Similar expression and localization of TbArl13 was also found in the mammal-infectious bloodstream form (BSF) cells (Fig. S4A,B). When using a protocol that efficiently extracts membrane-associated proteins such as the dually lipidated calflagin Tb24 (Tyler et al., 2009), we consistently observed a cytoskeletal pool for TbArl13 (Fig. S4C).

There are two major cytoskeletal structures in the trypanosome flagellum: the axoneme, which is nucleated from the basal bodies, and the paraflagellar rod (PFR), which only assembles after the flagellum exits the flagellar pocket and at a distance to the basal bodies (Bastin et al., 2000; Kohl et al., 1999; Fig. 1G). The TbArl13 signal began at precisely the same position as the axoneme marker YFP–RSP3 (Diener et al., 1993; Ralston et al., 2006), with the two signals overlapping completely until TbArl13 intensity diminished towards the distal end (Fig. 1D). The gradient-like distribution of TbArl13 along the axoneme was further illustrated by intensity plotting of both the anti-TbArl13 and radial spoke protein 3 (YFP–RSP3) signals (Fig. 1E). TbArl13, however, did not colocalize with the PFR, and a gap was evident between the proximal ends of PFR and TbArl13 (Fig. 1F, green and red arrows). TbArl13 was also readily observed in nascent flagella where no PFR had assembled (Fig. 1F, white arrowhead). Based on the above observations, we conclude that TbArl13 localizes to the flagellum and associates with the axoneme.

### The D/D domain is required for flagellar targeting and axonemal association of TbArl13

**Fig. 2.**
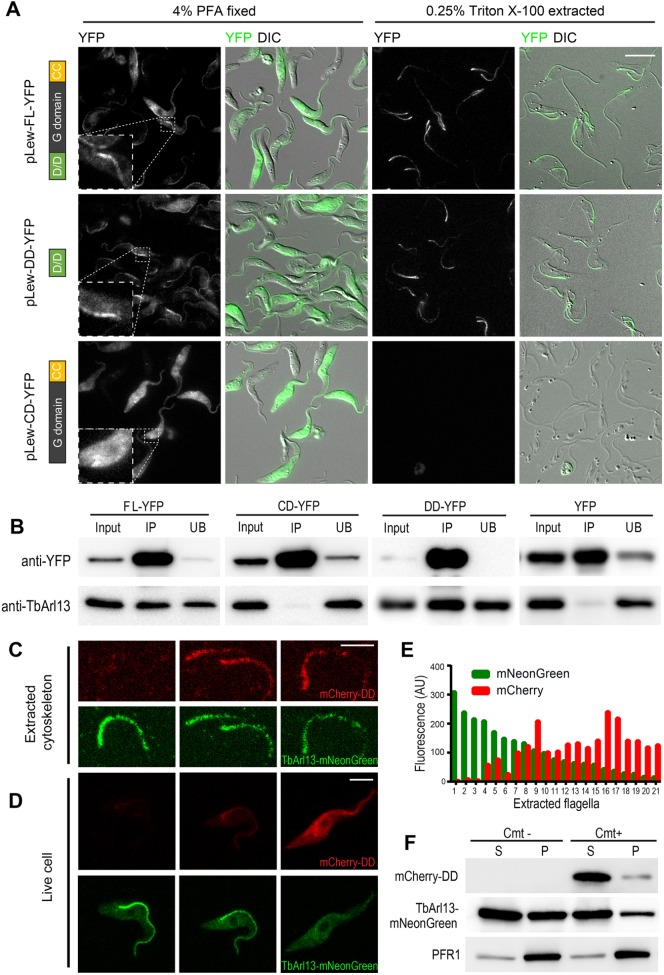
**TbArl13 is targeted to the flagellum via its D/D domain.** (A) Cells with inducible expression of YFP-tagged full length (FL–YFP) or truncated TbArl13 (DD–YFP, CD–YFP) were fixed with 4% PFA, with or without prior extraction with 0.25% Triton X-100. Insets focus on the proximal end of the flagellum of a representative cell, highlighting the enrichment of both FL–YFP and DD–YFP. (B) Co-immunoprecipitation of endogenous TbArl13 with FL–YFP, DD–YFP, but not with CD–YFP or YFP alone. GFP-nAb beads were incubated with lysates from cells expressing FL–YFP, DD–YFP, CD–YFP or YFP alone. Anti-TbArl13 antibody was used to detect the presence of native TbArl13 in input (10%), and the co-immunoprecipitated (IP) and unbound (UB) fractions. (C,D) Fluorescence images of 0.25% NP40 extracted (C) or live cells (D) with endogenous expression of TbArl13–mNeonGreen (green) and varying levels of mCherry–DD (red). (E) Quantification of mCherry–DD and TbArl13–mNeonGreen intensities in 21 extracted flagella such as those shown in C. AU, arbitrary units. (F) Cell fractionation assay using 1% NP40 shows a reduced level of TbArl13–mNeonGreen in the cytoskeletal fraction (P, pellet) when mCherry–DD expression is induced (Cmt+). The soluble fraction (S, supernatant) did not show observable changes. Scale bars: 5 μm.

The D/D domain was first characterized and best studied in the regulatory subunits of PKA (Lester and Scott, 1997; Rubin, 1994). The subcellular localizations of the PKA regulatory subunits are regulated through the docking of its D/D domain to A-kinase-associated proteins (AKAPs) found in various intracellular locations. To investigate whether the D/D domain in TbArl13 plays a role in its flagellar targeting, we generated stable cell lines containing tetracycline-inducible expression of YFP fused to full length (FL–YFP) and truncated TbArl13 (DD–YFP and CD–YFP). DD–YFP contained the D/D domain (amino acids 1–62), whereas CD–YFP spanned the GTPase domain and the CC motif (amino acids 61–270). All three recombinant proteins were found throughout the PFA-fixed cells, possibly due to overexpression, but only FL–YFP and DD–YFP demonstrated a clear enrichment at the flagellum, which was more obvious upon detergent extractions (Fig. 2A). In contrast, the CD–YFP signal was completely lost in detergent-extracted cells. Cell fractionation analyses also confirmed that both FL–YFP and DD–YFP had a detergent-resistant pool, while CD–YFP was completely soluble (Fig. S5A). These results demonstrate that the D/D domain fully accounts for the flagellum targeting and axonemal association of TbAr113.

Since the D/D domain is also known to mediate PKA regulatory subunit dimerization (Scott et al., 1990), we asked whether TbArl13 can self-associate. Co-immunoprecipitation (co-IP) assays showed that endogenous TbArl13 co-precipitated with both FL–YFP and DD–YFP but not with CD–YFP or YFP alone (Fig. 2B). These results suggest that TbArl13 self-associates through the D/D domain, and that the D/D domain of TbArl13 likely functions in the similar ways to the D/D domain of PKA.

If TbArl13 solely relies on the D/D-mediated axoneme docking for its flagellar targeting, inhibition of the docking process would not only disrupt the axoneme association but also its overall flagellar enrichment. We reasoned that excess amounts of D/D domains alone might compete with TbArl13 for docking sites, serving as an inhibitor of TbArl13 docking. To test this hypothesis, we overexpressed D/D domain (mCherry–DD) using a cumate (Cmt)-inducible system (Li et al., 2017) in cells expressing endogenously tagged TbArl13–mNeonGreen. In detergent-extracted cells, we consistently observed weaker flagellar mNeonGreen (Fig. 2C, quantifications shown in Fig. 2E and Fig. S5B) and reduced TbArl13–mNeonGreen in the cytoskeletal fraction (Fig. 2F) in cells expressing mCherry–DD. Remarkably, overall flagellar enrichment of TbArl13–mNeonGreen in live cells was also reduced (Fig. 2D). Similar results were obtained using 3xHA–DD, thereby ruling out possible fluorescence energy transfer effects between mNeonGreen and mCherry (data not shown). These results, therefore, support a diffusion-retention model for TbAr113 flagellar targeting, where TbArl13 enters the flagellum by diffusion but is retained in the flagellum by D/D-mediated axoneme docking (see Fig. 7). Furthermore, the number of TbArl13-D/D docking sites on the axoneme is likely limited.

Notably, overexpression of the TbArl13 D/D domain is not well tolerated by the cells as high expression levels could not be maintained for more than 2 days (data not shown). The dominant-negative effect of D/D overexpression in BSF cells will be discussed below, where it is more pronounced.

### TbArl13 is essential for flagellum biogenesis and cell survival

**Fig. 3.**
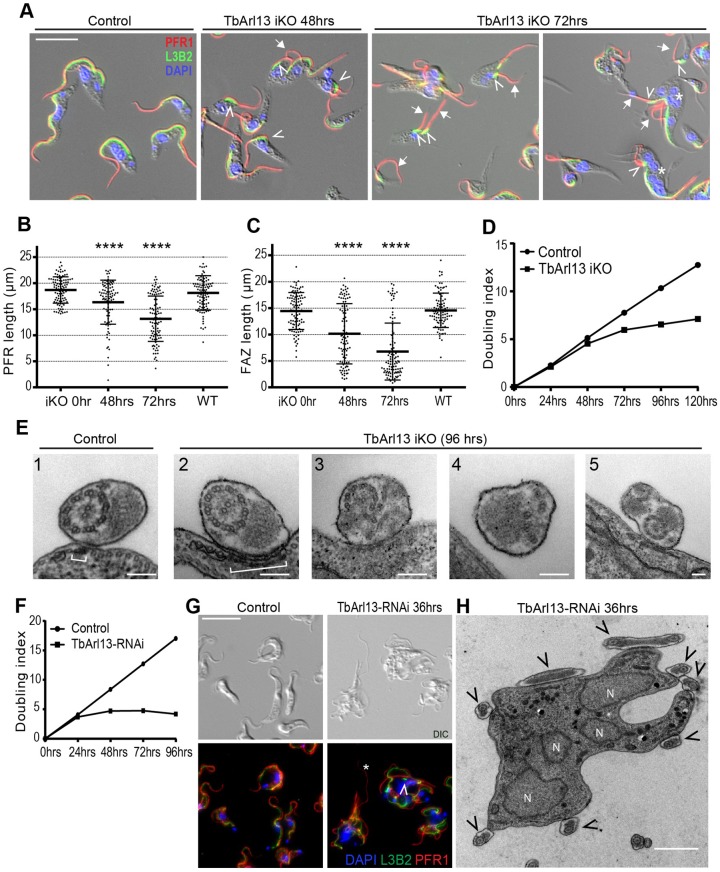
**TbArl13 is essential for flagellum biogenesis and cell survival.** (A) TbArl13 iKO cells at 0 h (control), 48 h and 72 h upon tetracycline removal were labeled with anti-PFR1 antibody to mark the PFR (red), L3B2 for the FAZ (green), and DAPI (blue). Cells with shortened FAZ (arrowheads) and detached/shortened flagella (arrows) are observed. Scale bar: 5 μm. (B,C) Statistical analyses of PFR and FAZ length measurements. Single flagellated cells were measured for each time point (*n*=99, 92, 100, 95 for 0 h, 48 h, 72 h and WT respectively), and the results are shown as mean±s.d. ****P<0.0001 [two tailed Student's *t*-test was used to evaluate the significance of difference between the test groups and the control group (iKO 0 h)]. (D) Growth curves of TbArl13 iKO and control cells. The growth curves shown are representative of two independent experiments. (E) Representative TEM images of flagellum from control and TbArl13 iKO cells (96 h post induction). Brackets: FAZ filaments. Scale bar: 100 nm. (F) TbArl13-RNAi leads to growth arrest in BSF cells. The growth curves shown are representative of two independent experiments. (G) BSF control and TbArl13-RNAi cells were fixed and labeled with anti-PFR1 antibody (red), L3B2 (green) and DAPI (blue). Asterisk, detached flagellum; arrowhead, shortened FAZ. (H) Representative TEM image of a TbArl13-RNAi-induced ‘monster’ cell with multiple nuclei (N) and flagella (arrowheads). Scale bar: 1 μm.

The cellular functions of TbArl13 were investigated by Arl13 inducible knockout (iKO) in PCF cells. Both endogenous TbArl13 alleles were replaced with drug resistance genes, and ectopic TbArl13–YFP expression was achieved through a tetracycline (Tet) inducible cassette stably incorporated into the genome (Fig. S6A). At 72 h after tetracycline washout, TbArl13 expression was not detectable (Fig. S6B). Unlike control cells that each contain single or duplicated flagella that tightly adhere to the cell body via the flagellum attachment zone (FAZ), TbArl13-iKO cells displayed severe flagellar defects (Fig. 3A, arrows, B). The flagella (marked by anti-PAR antibody to stain the PFR) became significantly shorter (from 18.7±0.25 μm in control cells to 13.2±0.44 μm in iKO cells, 72 h post induction; mean±s.e.m.). The FAZ (marked by L3B2) length also reduced dramatically (from 14.5±0.35 μm in control cells to 6. 8±0.54 μm in iKO cells, 72 h post induction), with some L3B2 signals appearing as condensed stubs at 72 h post iKO induction (Fig. 3A, arrowheads, C). Both flagella and the associated FAZ are key regulators in trypanosome cytokinesis (Kohl et al., 2003; Robinson et al., 1995; Vaughan et al., 2008; Zhou et al., 2011), and this may explain the appearance of multi-nuclear, multi-flagellated ‘monster cells’ in TbArl13-iKO population (Fig. 3A, asterisks). Consistent with this, TbArl13 iKO cells could not proliferate beyond 72 h (Fig. 3D).

**Fig. 4.**
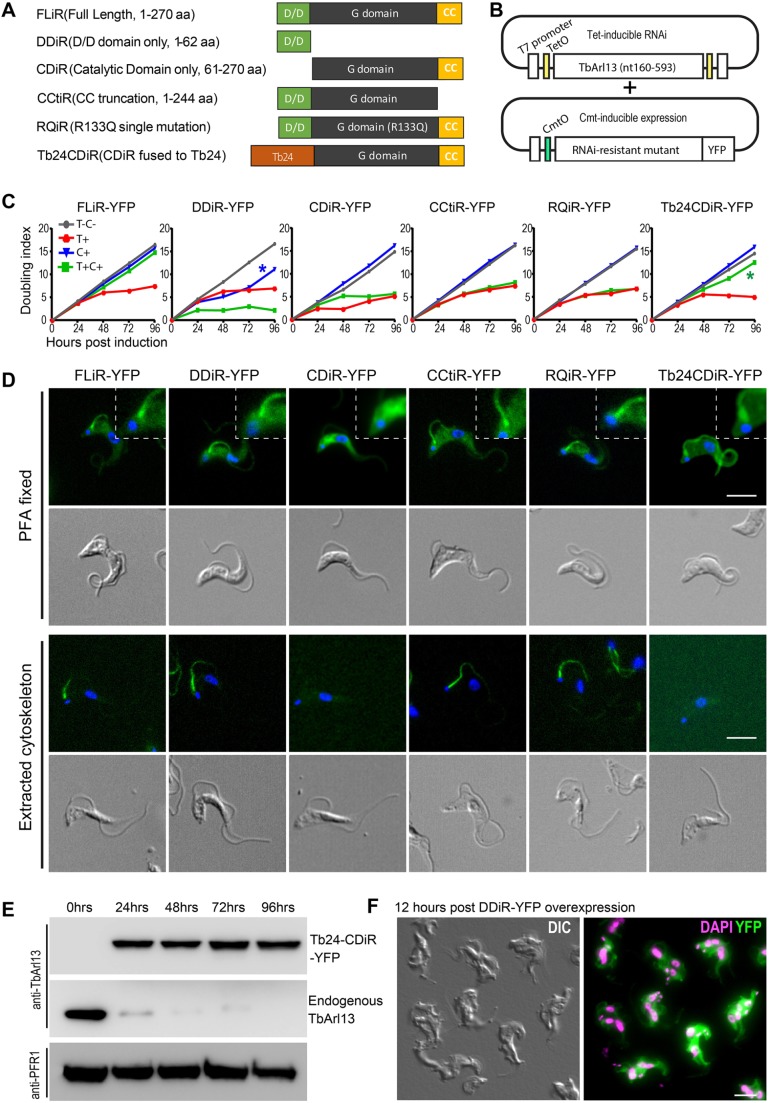
**Flagellar enrichment of TbArl13 catalytic domain is critical for cell proliferation.** (A,B) Schematic presentation of the TbArl13 truncations and mutants (A) used in the RNAi complementation experiments (B). TbArl13-RNAi is controlled by a tetracycline-inducible system and expression of RNAi-resistant (iR) mutants is controlled by a cumate-inducible system. (C) Proliferation of TbArl13-RNAi cells carrying different RNAi resistant mutants under indicated culture conditions were plotted. T-C-: control without tetracycline or cumate addition. T+: tetracycline only (10 μg/ml). C+: cumate only (2 μg/ml). T+C+: tetracycline (10 μg/ml) and cumate (2 μg/ml). Blue asterisk: initial growth inhibition and later recovery upon DDiR–YFP induction. Green asterisk: sustained proliferation of Tb24–CDiR–YFP-expressing cells under TbArl13-RNAi. The growth curves shown are representative of two independent experiments. (D) BSF cells stably expressing the indicated mutant constructs were fixed with 4% PFA before (whole cell) or after detergent extraction (cytoskeleton), stained with DAPI and imaged by fluorescence microscopy. Insets show enlarged areas at the base of the flagellum, highlighting the flagellum enrichment of FLiR–YFP, DDiR–YFP, CCtiR–YFP, RQiR–YFP and Tb24CDiR–YFP, and the lack of flagellum enrichment for CDiR–YFP. All D/D domain-containing mutants were resistant to detergent extraction. (E) Immunoblots confirmed cumate-induced expression of Tb24CDiR–YFP and tetracycline-induced depletion of endogenous TbArl13. Both Tb24CDiR–YFP and endogenous TbArl13 were probed with anti-TbArl13 antibody. Anti-PFR1 antibody was used as a loading control. (F) A representative image showing cytokinesis arrest in cells induced for DDiR–YFP overexpression (12 h after addition of 30 μg/ml cumate). Scale bars: 5 μm.

To find out whether the shortening of flagella was associated with ultrastructural defects, TbArl13 iKO cells were examined using transmission electron microscopy (TEM) (Fig. 4E). For flagella (or regions of flagella) that were still attached to the cell body, the associated FAZ filament appeared much wider in iKO cells than in wild-type (WT) cells (Fig. 3E, brackets). This observation is consistent with the condensed appearance of FAZ stubs marked by L3B2 staining (compare with Fig. 3A). Strikingly, flagella with severe ultrastructural defects could be seen in the iKO cells but never in control (Fig. 3E). The defects were heterogenous – both axonemes and the PFR could be malformed, with the axonemal defects ranging from misplaced microtubule doublets to total disruption of the 9+2 pattern. Occasionally, two axonemes could be observed in the same flagellar lumen. These wide-ranging phenotypes suggest that the loss of TbArl13 results in total derangement of flagellum biogenesis.

We also studied the TbArl13-knockdown phenotypes in the BSF cells. TbArl13-RNAi completely arrested BSF cell proliferation within 48 h of induction (Fig. 3F; Fig. S6C), generating ‘monster cells’ with multiple flagella and nuclei, indicative of cytokinesis failure (Fig. 3G,H). Despite occasional flagellum detachment and FAZ shortening (Fig. 3G, arrowhead and asterisk), we did not observe any major change to flagellar length, nor gross perturbation in flagellar ultrastructure as seen previously in the PCF cells. It has long been noticed that cytokinesis and cell survival of BSF cells are much more sensitive to flagellum perturbation than in the PCF (Broadhead et al., 2006; Ralston and Hill, 2006; Wheeler et al., 2013). The acute cytokinesis arrest and cell death in BSF following TbArl13 knockdown may have prevented the progressive manifestation of flagellar defects as seen in PCF cells. Taken together, these results indicate that TbArl13 is an essential protein in *T. brucei* and is a critical player in flagellum biogenesis.

### Flagellar enrichment of TbArl13 is crucial for its cellular functions

We next investigated whether the function of TbArl13 depends on its distinct flagellar targeting. To this end, we devised a double-inducible RNAi complementation assay in BSF cells. In this assay, Cmt-inducible expression vectors containing different RNAi-resistant (iR) TbArl13 mutants were stably transfected into BSF cells with stable integration of Tet-inducible TbArl13-RNAi (Fig. 4A,B).

As shown in Fig. 4C, the expression of full-length RNAi-resistant TbArl13 (FLiR–YFP) was able to restore cell proliferation when native TbArl13 was depleted via RNAi, thereby validating the experiment setup and confirming the specificity of TbArl13-RNAi phenotypes described above. Expression of the R133Q mutant (RQiR–YFP), which corresponds to the disease associated R79Q mutation in human Arl13b, could not rescue TbArl13-RNAi. The CC domain, which has been shown to be involved in the GEF activity of Arl13b (Gotthardt et al., 2015), is also indispensable as CCtiR–YFP failed to rescue the growth of TbArl13-RNAi cells. Consistent with results observed in PCF cells (see Fig. 2A), CDiR–YFP did not show enrichment in the flagellum nor association with the axoneme, and could not rescue TbArl13-RNAi in BSF. These results suggest that D/D-mediated flagellar targeting is required for the cellular functions of TbArl13.

From the above experiments, it remained unclear whether D/D had any functional role beyond flagellar targeting. Additionally, it was not clear whether flagellar enrichment and axoneme association are both required for TbArl13 functions, since D/D deletion abolished both attributes. To address these issues, we generated a chimeric protein (Tb24CDiR) by swapping the D/D domain of TbArl13 with the entire sequence of the well-established flagellar membrane-associated protein calflagin Tb24 (Tyler et al., 2009). As expected, Tb24CDiR–YFP exhibited a flagellar membrane localization and could be extracted by detergent, both features distinct to wild type TbArl13 (Fig. 4D; Fig. S6D). Strikingly, Tb24CDiR–YFP was able to support continued cell proliferation in the absence of endogenous TbArl13 (Fig. 4C,E). These results indicate that the flagellar localization of the catalytic domain of TbArl13 (GTPase domain and CC motif) is required to support BSF cell proliferation. However, the exact location of the protein, whether membrane- or cytoskeleton-associated, and the mechanisms through which flagellar enrichment is achieved could be flexible.

Interestingly, we found that DDiR–YFP expression alone had an inhibitory effect on cell proliferation before expression was lost and growth recovered (Fig. 4C, blue asterisk). Microscopy data showed rapid accumulation of ‘monster cells’ at a higher induction level (15 µg/ml instead of 2 µg/ml) (Fig. 4F). Combined with our earlier finding showing that DD–YFP overexpression displaces endogenous TbArl13 (Fig. 2C–F), this finding lends further support to our conclusion that flagellar enrichment of TbArl13 is critical in *T. brucei*.

### TbArl13 catalyzes both TbArl3A and TbArl3C

To investigate the interaction network of TbArl13, we performed proximity-dependent biotin identification (BioID) of potential TbArl13-interacting partners (see Materials and Methods for details). Specific protein hits were ranked based on their relative protein content (Table 1). Notably, two Arl3 homologs named TbArl3C and TbArl3A were the fourth and seventh most abundant proteins identified through BioID. Furthermore, a protein (Tb927.10.5810) homologous to the known Arl3 effector BARTL1 (Lokaj et al., 2015) was also found. The BioID results suggest that The Arl13b–Arl3 pathway is likely to be conserved in *T. brucei*.

**Table 1.**
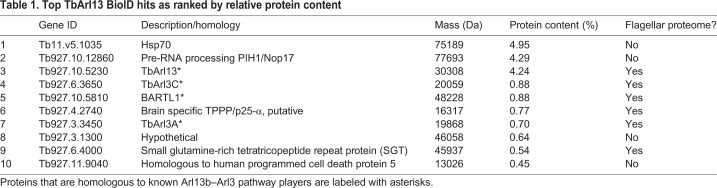
**Top TbArl13 BioID hits as ranked by relative protein content**

Three Arl3 homologs have been predicted in *T. brucei* (Cuvillier et al., 2000; Price et al., 2005) (Fig. S2). The localization of all three Arl3 homologs were first screened by overexpressing YFP-tagged recombinant proteins. TbArl3A–YFP was enriched at the nucleus and along the flagellum, as well as forming a punctum at the base of the flagellum (Fig. S7A). TbArl3C–YFP was highly concentrated at the base of the flagellum, with a weaker presence along the flagellum. TbArl3B–YFP, however, appeared to be completely excluded from the flagellum, and adopted a reticulated pattern that closely resembled the PCF mitochondrion (Povelones et al., 2013).

**Fig. 5.**
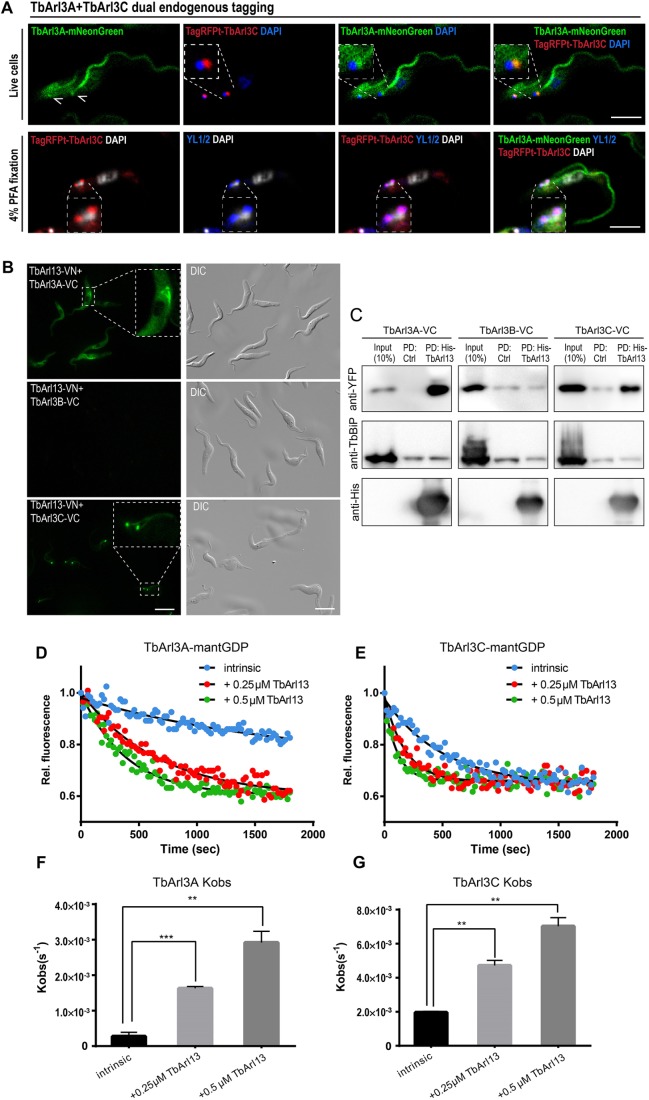
**TbArl13 interacts with two differentially localized Arl3 homologs.** (A) Cells co-expressing TbArl3A–mNeonGreen and TagRFPt–TbArl3C, both endogenously tagged, were imaged live (upper panel) or after 4% PFA fixation (lower panel). Kinetoplast and nuclear DNA were stained by cell-permeable DAPI. The YL1/2 antibody was used to mark the basal bodies (arrowheads). Scale bars: 5 μm. (B) Live PCF cells co-expressing TbArl13-VN and TbArl3A-VC (top), TbArl3B-VC (middle) or TbArl3C-VC (bottom) were imaged using fluorescence microscopy. Insets display enlarged regions exhibiting BiFC. Scale bars: 10 μm. (C) His–TbArl13-coated beads or control beads were able to pulldown (PD) TbArl3A- and TbArl3C-VC, but not TbArl3B-VC (all labeled by anti-YFP antibody). Anti-TbBiP was used as a control for non-specific binding to the beads. (D,E) TbArl13 accelerates dissociation of mant-GDP from both TbArl3A and TbArl3C, after the addition of unlabeled GTP at *t*=0 s. (F,G) Quantification (mean±s.e.m.) and statistical analysis of *K*_obs_ derived from results as shown in D and E. Experiments were replicated four times (*n*=4) for TbArl3A, and three times (*n*=3) for TbArl3C. **P<0.01, ***P<0.001 (two-tailed Student's *t*-test).

The differential localization of TbArl3A and TbArl3C was further validated by endogenously tagging both Arl3 genes in the same cell line for direct comparison (Fig. 5A). While TagRFPt–TbArl3C localized almost exclusively at the basal bodies, TbArl3A–mNeonGreen was additionally present and enriched along the flagellum. The nuclear localization of TbArl3A–YFP seen under overexpression conditions was not evident in cells with endogenously expressed TbArl3A–mNeonGreen, and therefore could be due to overexpression.

Bi-fluorescence complementation (BiFC; Kodama and Hu, 2010) was employed to study the interaction between TbArl13 and TbArl3 variants *in vivo* (Fig. S7B). Consistent with the BioID results, only TbArl3A–VC and TbArl3C–VC exhibited positive interaction with TbArl13–VN, allowing recombined Venus fluorescence to be detected both at the base and along the flagellum (Fig. 5B). Additionally, bacterially produced His–TbArl13 could pulldown TbArl3A–VC and TbArl3C–VC, but not TbArl3B–VC from *T. brucei* cell lysates (Fig. 5C). Reciprocal *in vitro* pulldown experiments also confirmed direct interaction between His-TbArl13 and both GST–TbArl3A and GST–TbArl3C (Fig. S7C,D).

Arl13b has been shown to be a GEF for Arl3. To investigate whether both TbArl3A and TbArl3C could be regulated by TbArl13 in the same fashion, we performed GEF assays using fluorescent, mant-labeled nucleotides (Gotthardt et al., 2015; Zhang et al., 2016). His–TbArl13 greatly accelerated mantGDP dissociation from both TbArl3A and TbArl3C, in the presence of excess unlabeled GTP (Fig. 5D–G). His-TbArl13 also accelerated the association of TbArl3A and TbArl3C to mantGTP (Fig. S7E,F). Interestingly, TbArl3C seems to have a much higher intrinsic nucleotide exchange rate compared to TbArl3A, further suggesting that the two Arl3 homologs may be regulated and/or function differentially. Bacterially produced GST–TbArl3B was not soluble in our hands. Considering that TbArl3B did not interact with TbArl13 in BiFC and *in vivo* pulldown assays, it is unlikely that it is an interacting partner of TbArl13 and was thus not included in the GEF activity assays.

### Both TbArl3A and TbArl3C function in flagellum biogenesis

**Fig. 6.**
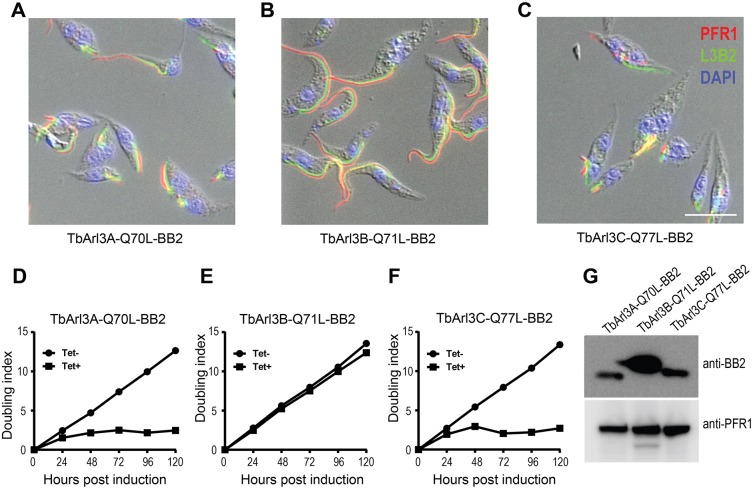
**TbArl3A and TbArl3C are critically involved in flagellum biogenesis.** (A–C) Inducible expression of the GTP-locked mutant forms of TbArl3A (Q70L) and TbArl3C (Q77L) both severely inhibited flagellum biogenesis in PCF cells. TbArl3B (Q71L), however, has no detectable effects. Anti-PFR1 and L3B2 antibody were used to mark the flagella and FAZ, respectively. Scale bar: 10 μm. (D–F) Growth curves of cells in the absence (Tet−) or presence (Tet+) of tetracycline for inducible expression of TbArl3 mutants. Growth curves shown are representative of two independent experiments. (G) Immunoblots confirm the expression of all three mutant TbArl13 homologs.

To investigate whether TbArl3A and TbArl3C have flagellar functions, we generated classic GTP-locked mutants by altering the catalytic glutamine residues at Switch 2 region to a leucine residue. Inducible expression of TbArl3A-Q70L–BB2 and TbArl3C-Q77L–BB2 both drastically inhibited flagellum biogenesis, with both flagellum and FAZ markers reduced into stubs or dots in most cells at 20 h post induction (Fig. 6A,C). In stark contrast, expression of TbArl3B-Q71L–BB2 had no observable impact on flagellum or cell morphology (Fig. 6B). In agreement with the flagellar defects, expression of TbArl3A-Q70L–BB2 or TbArl3C-Q77L–BB2 both inhibited cell proliferation. TbArl3B-Q71L–BB2 had no adverse impact on cell survival in culture (Fig. 6D–F), despite a higher expression level compared to the other two mutants (Fig. 6G). These results demonstrated that only TbArl3A and TbArl3C, both of which interact with TbArl13, are functionally relevant in flagellum biogenesis.

## DISCUSSION

Here, we report on a highly unusual Arl13b ortholog in the protozoan parasite *T. brucei*. Multiple lines of evidence support that TbArl13 is a bona fide Arl13b ortholog. At the sequence level, the catalytic domain is highly conserved among TbArl13 and other Arl13b orthologs. At the cellular level, TbArl13 localizes to the flagellum and is critical for flagellar biogenesis and functions. At the molecular mechanism level, TbArl13 catalyzes guanine nucleotide exchange in trypanosome Arl3 homologs. Trypanosomes (euglenozoan) are one of the most divergent groups of extant eukaryotes (Cavalier-Smith, 2010). These highly conserved attributes firmly support the idea that the role of Arl13b and the Arl13b–Arl3 pathway in ciliogenesis was established early in evolution.

Unlike the predominant flagellar membrane localization of Arl13b in animal cells, TbArl13 associates with the axoneme, along which it forms a gradient-like pattern. Interestingly, ARL-13 in *C. elegans* is also highly enriched at the proximal segment in amphid and phasmid cilia, albeit on the membrane domain (Cevik et al., 2010, 2013). The same studies also found proximal enrichment of mammalian Arl13b in the cilia of MDCKII, mouse oviduct and tracheal epithelial cells. It is striking that fundamentally different flagellar-targeting mechanisms, which target different sub-flagellar domains, result in a similar Arl13b pattern along the flagellum/cilium. This appears to be a case of convergent evolution, where selective pressure favors strong Arl13b presence at the proximal end of cilia. A recent study suggests that after Arl3-GTP-dependent release, ciliary cargos (INPP5E) may be relayed onto the IFT machinery for trafficking within the cilium (Kosling et al., 2018). In this perspective, one advantage that the asymmetrical distribution of the Arl3-GEF could provide is the maximization of cargo release efficiency near the ciliary gate (where it is picked up by IFT). However, this advantage is likely not essential, as suggested by our rescue results and the observations that not all cell types show such proximal Arl13b enrichment at the cilia.

**Fig. 7.**
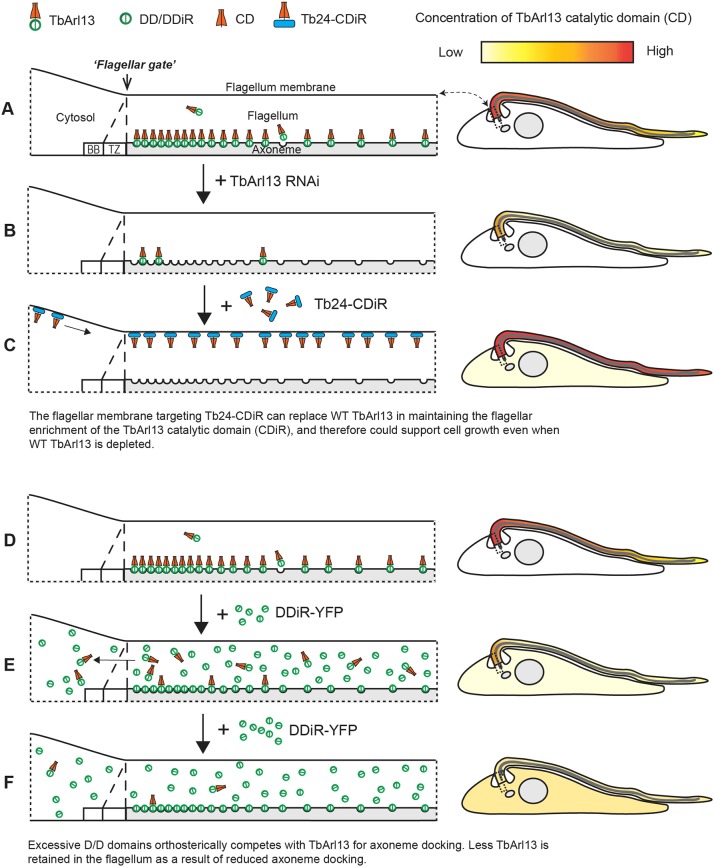
**A diffusion-retention model accounts for flagellar enrichment of TbArl13, which is critical for TbArl13 cellular functions.** (A,D) In wild-type cells, TbArl13 is specifically enriched in the flagellum by means of axoneme docking through the D/D domain (Figs 1 and 2), possibly via its interaction with a putative axonemal docking partner. BB, basal body; TZ, transition zone. (B) TbArl13-RNAi is lethal for the cell (Fig. 3). (C) Expression of Tb24–CDiR–YFP could rescue cell growth in the absence of endogenous TbArl13 (Fig. 4), because the flagellar enrichment of the catalytic domain of TbArl13 is restored through Tb24 (Fig. 4 and Fig. S6D). (E,F) As the D/D domain is sufficient for axonemal docking, excess amounts of DDiR–YFP compete with native TbArl13 for limited docking sites (Fig. 2). As more TbArl13 molecules diffuse out of the flagellum, overall flagellar enrichment of TbArl13 is compromised and results in a dominant-negative effect.

We have shown that the axonemal association of TbArl13 is mediated by its D/D domain, which is conserved within the kinetoplastid group but absent in other eukaryotes. D/D domains were first described and are best studied in the targeting of mammalian PKA. However, accumulating evidence (including this study) suggest that they are found in a wide range of non-PKA proteins including some flagellar proteins (Carr et al., 2001; Fujita et al., 2000; Sivadas et al., 2012). Thus, D/D domains may represent a class of modular, generally adaptable domain for subcellular protein targeting. Based on the observation that the overexpressed D/D domain displaced TbArl13–mNeonGreen from the axoneme and also weakened its overall flagellar enrichment, we propose that a ‘diffusion-retention’ model can account for the flagellar targeting/enrichment of TbArl13 (Fig. 7). In this model, TbArl13 could freely diffuse between the cytoplasm and flagellar lumen but is predominantly enriched in the flagellum due to its affinity with the axoneme. This affinity of TbArl13 to the axoneme is conferred by the D/D domain, likely through specific interaction with a putative axonemal docking partner. Indeed, while ciliary membrane protein entry is tightly regulated by a barrier system including the transition zone complex and a septin ring (Hu et al., 2010; Reiter et al., 2012), recent studies suggest that the ‘ciliary gate’ for soluble proteins is more ‘leaky’ and may grant ciliary access to proteins up to 100 kDa (Breslow et al., 2013) or even 650 kDa (Lin et al., 2013). Therefore, the diffusion-retention mechanism may play a significant role in the ciliary targeting of smaller soluble proteins (Nachury et al., 2010).

Taking advantage of this simple D/D-mediated flagellar-targeting mechanism, we explored the relationship between the localization and functions of TbArl13. The chimeric Tb24CDiR–YFP, which could enrich on the flagellum membrane but did not form a gradient distribution along the flagellum, could functionally replace WT TbArl13. By contrast, CDiR–YFP, which could enter the flagellum but showed no enrichment compared to the cytoplasm, could not rescue TbArl13-RNAi cells. These results suggest that neither the axoneme association nor the gradient-like distribution is essential for TbArl13 function. Instead, flagellar enrichment of the TbArl13 catalytic domain, via the D/D domain or Tb24, is required (Fig. 7A–C). The flagellar enrichment of TbArl13 may be crucial for its function as an Arl3-GEF, generating a greater concentration of Arl3(A/C)-GTP inside of the cilia, thereby facilitating directional transport of ciliary cargoes (Fansa et al., 2016; Gotthardt et al., 2015; Ismail et al., 2011). A recent study showed that a myristoylated (instead of palmitoylated) Arl13b mutant can localize to the cilia but cannot fully complement the loss of endogenous Arl13b functions (Roy et al., 2017). We nevertheless noted that the myristoylated mutant outperformed all other non-ciliary-localized Arl13b mutants tested in rescuing the ciliary biogenesis defects both in Arl13b-null cells and fish. Additionally, the poor protein stability of this Arl13b mutant could have been a major factor preventing a full rescue.

The dominant-negative effects of expressing just the D/D domain could be due to its activity as an orthosteric inhibitor, as these domain truncations can compete with WT TbArl13 for docking partners on the axoneme (Figs 2C–F and 7D–F). Since D/D-mediated Arl13b targeting is not found in other eukaryotes except for the kinetoplastids, this docking machinery presents an attractive therapeutic target for a range of human and animal diseases caused by this group of parasites. Indeed, the D/D–AKAP interaction in the PKA pathway is known to be susceptible to interference with short peptides, peptidomimetic or small molecules, and is being explored as a potential therapeutic target (Christian et al., 2011; Hundsrucker and Klussmann, 2008; Tröger et al., 2012; Wang et al., 2014).

TbArl13 likely dimerizes due to the D/D domain. Interestingly, it was reported that human Arl13b (which does not possess a D/D domain) could also self-associate through the ‘N-terminal domain’ (Hori et al., 2008). A later biochemical study of CrArl13b provided strong evidence against Arl13b dimerization, but the recombinant protein used in the study appeared to be a truncation that lacks residues 1–17 (Miertzschke et al., 2014). If the extreme N-terminal residues in animal Arl13b indeed facilitate dimerization, Arl13b dimerization would merit further study. Regardless, Arl13b dimerization is likely not required for Arl3 GEF activity, since the truncated CrArl13b was used to first characterize the GEF activity (Gotthardt et al., 2015). Similarly, our positive rescue results with Tb24CDiR–YFP suggest that dimerization via D/D is not obligatory. It is possible that Tb24 might form dimers, although current evidence is inconclusive (Xu et al., 2012).

Although Arl3 has not been found to be associated with any ciliopathies, classic ciliopathy manifestations have been reported in Arl3 knockout and Arl3 conditional knockout mice (Hanke-Gogokhia et al., 2016; Schrick et al., 2006). The first evidence suggesting a ciliary function for Arl3 came from pioneering research done in the trypanosomes, where the expression of the GTP-locked form of Arl3A severely inhibited flagellum biogenesis in several species (Cuvillier et al., 2000; Sahin et al., 2004). Here, we show that expression of GTP-locked forms of TbArl3A and TbArl3C both inhibited flagellum biogenesis. TbArl13 binds and exhibits GEF activity to both TbArl3A and TbArl3C, while TbArl3C has much higher intrinsic nucleotide exchange activity than TbArl3A. Interestingly, TbArl3A and TbArl3C adopt different localizations, with TbArl3A featured prominently along the flagellum and TbArl3C highly enriched on the basal body. The previously annotated TbArl3B (Cuvillier et al., 2000; Price et al., 2005) did not interact with TbArl13 nor did it cause any observable phenotypes when its GTP-locked form was expressed. Taken together with the observation that it did not fall under the Arl3 clade in our phylogenic analysis (Fig. S1B), it is uncertain whether TbArl3B is a true Arl3 ortholog. Our results suggest a possible expansion and/or specialization of Arl3 functions in *T. brucei*. Considering the well-established role of Arl3 as a cargo displacement factor, an intriguing scenario is that the differential localization of the two TbArl3 proteins allows for different cargos to be released at different flagellar locations. Mammalian cells have only one Arl3, which is found in multiple structures including the centrioles (or basal bodies of cilia), the cilia and the Golgi (Grayson et al., 2002; Zhou et al., 2006). It is possible that in higher eukaryotes, Arl3 cargos are also location dependent and this may help to explain its multiple roles during ciliogenesis (Hanke-Gogokhia et al., 2016). Further dissection of TbArl3A and TbArl3C functions could shed more light into the complex role of Arl3 in cilia biology.

## MATERIALS AND METHODS

### Bioinformatics

Putative Arf/Arl GTPases were retrieved by searching ARF domain (Domain ID: IPR024156) in the Tritryp Database (http://tritrypdb.org) (Aslett et al., 2010). Large proteins (>40 kDa) were removed from the list. Sequences of Arf/Arl family members from other eukaryotic model systems were retrieved from NCBI GenBank (Benson et al., 2013). Phylogenetic analysis was carried out using both Maximum Likelihood and Bayesian phylogenetic inferences. Initial sequence alignment was carried out in mCoffee (Wallace et al., 2006), based on which the Arf/Arl GTPase domains were extracted for downstream analyses. The GTPase domains were re-aligned using MAFFT-LiNSI (v. 7.394) (Katoh and Standley, 2013). Alignment was next masked using ALISCORE (v. 2.0) (Kück et al., 2010; Misof and Misof, 2009) and ALICUT (v. 2.31; https://github.com/PatrickKueck/AliCUT) to remove the positions that were not unambiguously aligned. Both Maximum Likelihood (ML) and Bayesian phylogenetic inferences were conducted on the resulting alignment. Model testing was performed with ProtTest 3 (v. 3.4.2) (Darriba et al., 2011). The best fit model was selected based on the Akaike Information Criterion (AIC). Maximum Likelihood inference was carried out using RaxML (v. 8.2.10) (Stamatakis, 2014), where the best scoring tree was obtained using twenty alternative runs on distinct starting trees. Multiparametric bootstrapping was carried with automatic bootstrapping option (autoMRE). Bayesian phylogenetic inference was conducted using MrBayes (v. 3.2.6) (Ronquist and Huelsenbeck, 2003). Two parallel analyses were run for 10×10^6^ generations with four chains each. Sampling frequency was set at 1000 and initial 10^3^ trees were discarded as burn-in. A consensus of the two trees was obtained and the convergence was assessed using Tracer (v. 1.6) (Rambaut et al., 2018).

Identity and similarity scores were calculated and tabulated using the SIAS program (imed.med.ucm.es/Tools/sias.html). Multiple sequence alignments were visualized using Sequence Manipulation Suite (Stothard, 2000). The D/D domain in TbArl13 was identified using NCBI Conserved Domains and Interpro (https://www.ebi.ac.uk/interpro/). The COILS server was used for coiled-coil prediction (Lupas et al., 1991). Palmitoylation prediction was conducted using NBA-Palm (Xue et al., 2006).

### Cell culture and transfection

All PCF cells were maintained in Cunningham medium supplemented with 15% heat-inactivated fetal bovine serum (Hyclone) at 28°C. All BSF cells were maintained in HMI-9 medium containing 10% heat-inactivated fetal bovine serum (Hyclone) at 37°C with 5% CO_2_. Cell transfections were carried out based on published protocols (Beverley and Clayton, 1993). *Trypanosoma brucei rhodesiense*, YTat1.1 (Ruben et al., 1983) cells stably engineered for tetracycline (Tet) and/or cumate (Cmt) double-inducible expression (Poon, 2012) were used for TbArl13 inducible knockout, and all endogenous tagging and inducible expression experiments in the procyclic form. TbArl13-RNAi cells were generated in procyclic *Trypanosoma brucei brucei*, 29.13 cells (Wirtz et al., 1999). For BSF experiments, phenotypes of TbArl13-RNAi and rescue assays were analyzed using Dlb427 (*T. brucei* monomorphic Lister 427 cells engineered for Tet/Cmt double-inducible expression) (Li et al., 2017; Poon, 2012). A PCR-based endogenous tagging system (pPOT) was adopted to generate TbArl13–mNeonGreen, TbArl3A–mNeonGreen and TagRFPt–TbArl3C endogenous tagging (Dean et al., 2015).

### Plasmids and antibodies

Plasmids used in this study are listed in the supplementary information (Table S1). Point mutations were introduced into WT TbArl13 or TbArl3 genes by site-directed mutagenesis using inverse PCR (Reikofski and Tao, 1992). For TbArl13-RNAi complementation assays, codons in the full-length TbArl13 coding sequence (CDS) were replaced with alternative codons whenever applicable to generate an RNAi-resistant version (iR) of TbArl13 CDS. The synthesized RNAi-resistant CDS served as PCR template from which different mutants/truncations were generated.

Rabbit anti-TbArl13 polyclonal antiserum was generated using purified His–TbArl13 expressed in *E. coli*. Antiserum was further affinity purified on PVDF membrane strips loaded with GST–TbArl13 (Ritter, 1991). Other antibodies used in this study are listed in the supplementary information (Table S2).

### Fluorescence and electron microscopy

Unless otherwise stated, PCF and BSF cells were fixed with 4% paraformaldehyde before or after extraction with 0.25% NP-40 or Triton X-100. The fixed cells or cytoskeleton samples were blocked with 3% BSA and then incubated with primary and secondary antibodies. Images were captured either on a Zeiss Axio Observer Z1 fluorescence microscope with a CoolSNAP HQ2 CCD camera (Photometrics) or on an Olympus FLUOVIEW FV3000 confocal microscope.

For TEM, PCF and BSF cells were fixed and resin-embedded based on established protocols (Höög et al., 2010). Ultra-thin sections were post-stained with Reynold's lead citrate for 10 min. Transmission electron microscopy was performed using a JEM-1220 TEM from JEOL Asia, and an Orius 830 CCD camera (2K by 2K) from Gatan.

### Image analysis

All images were adjusted and edited for presentation using ImageJ. For all images subject to measurement and comparison, both acquisition and image display parameters were kept constant. For flagellar fluorescence intensity quantification, axonemes were traced using freehand tool in ImageJ (line width=3 pixels). The average intensity values along the first 2 µm of the anterior axonemes were used for analysis in Fig. 2E and Fig. S5B. The anterior tip of the axonemes were determined using either the beginning of mCherry or mNeonGreen signals, whichever was stronger. A correlation coefficient was calculated using Graphpad Prism 6.

### Bimolecular fluorescence complementation

VN and VC fragments were cloned from pBiFC-VN155 (I152L) (Addgene plasmid #27097; Kodama and Hu, 2010) and pCE-BiFC-VC155 (Addgene plasmid #22020; Shyu et al., 2008). BiFC reporter plasmids were generated by fusing TbArl13 to VN in the Tet-inducible pLEW100 vector, or TbArl3 coding sequences to VC in the Cmt-inducible pDEX vector. Procyclic cells stably transfected with both VN- and VC-containing plasmids were induced with cumate (10 µg/ml) and tetracycline (20 µg/ml), alone or together for 24 h before live fluorescence imaging using YFP parameters.

### Proximity-dependent biotin identification

PCF cells stably transfected with pDEX-FLiR-MycBirA were induced with 10 µg/ml cumate for 24 h. These cells and WT PCF cells (negative control) were then cultured in the presence of 50 µM biotin for an additional 24 h. The procedures for streptavidin-based pulldown of biotinylated proteins following a published protocol (Morriswood et al., 2013). To maximize peptide recovery, captured proteins were reduced, alkylated and digested on the streptavidin-coated beads. LC-MS/MS of digested peptides were carried out on an Eksigent nanoLC Ultra and ChiPLC-nanoflex (Eksigent, Dublin, CA) in Trap Elute configuration. Tandem MS analysis was performed on a TripleTOF 5600 system (AB SCIEX, Foster City, CA, USA) in Information Dependent Mode. MS spectra were acquired using the Analyst 1.6 software (AB SCIEX). Protein identification was performed with MASCOT Server 2.4.0 (Matrix Science), and data was searched against the *T. brucei* protein database (TbruceiTREU927, Release-28). Protein identification threshold was set at 1% false discovery rate (FDR) based on reversed protein sequences. ‘Protein content’ was calculated using the emPAI method (Ishihama et al., 2005), which estimates the weight fraction of each protein against the weight of the total pulldown mixture. Protein IDs that were found only in the TbArl13-mycBirA sample but not in the negative control sample were kept and ranked based on their relative protein content. To determine whether a protein is in the ‘flagellar proteome’, the result list was compared with past proteomics studies on *T. brucei* flagella (Broadhead et al., 2006; Oberholzer et al., 2011; Subota et al., 2014) using the Tritryp Strategy tool (tritrypdb.org).

### Pulldowns and co-IP experiments

Reciprocal *in vitro* pulldowns of purified His–TbArl13 and GST–TbArl3A/C beads were carried out through standard protocols (Einarson et al., 2007) and were analyzed by SDS-PAGE followed by Coomassie Brilliant Blue staining. For pulldown of TbArl3A/B/C-VC from *T. brucei* lysates, His–TbArl13-coated beads were incubated with lysates of cells expressing the respective transgenes for 2 h at room temperature, in the presence of 15 µM imidazole. As negative controls, Ni-NTA beads were incubated with untransformed BL21 bacterial lysate before incubating with the same cell lysates under same conditions. After incubation, beads were washed three times with PBS containing 20 µM imidazole and once with PBS. Bound proteins were eluted by boiling in Laemmli buffer. Co-immunoprecipitation studies of TbArl13 with FL-YFP, DD-YFP, CD-YFP and YFP only were conducted using GFP-nAb beads (Allele Biotechnology) following the manufacturer's protocol.

### Guanine nucleotide exchange assay and GTP-binding assay

Guanine nucleotide exchange assays were conducted based on established protocols (Gotthardt et al., 2015). Briefly, GST–TbArl3A and GST–TbArl3C were preloaded with mant-GDP in the presence of 50 mM EDTA and 5-fold mant-GDP (Sigma) at 20°C for 90 min. The nucleotide change was stopped by adding 100 mM MgCl_2_. Excess nucleotides were removed by desalting columns (7K MWCO; Thermo Scientific). 0.5 μM mant-GDP-labeled GST–Arl3A or GST–Arl3C were incubated with different concentrations of His–TbArl13 and unlabeled GTP (20-fold excess) at 20°C. The GTP-binding assay was performed using the RhoGEF exchange assay kit (Cytoskeleton, Inc) based on the manufacturer's instructions. 4 μM purified GST–Arl3A or GST–Arl3C were incubated with or without 1.5 μM His–TbArl13 in the presence of 1.5 μM mant-GTP at 20°C. All fluorescence data was recorded with a Tecan Infinite M200 plate reader. Both GEF and GTP-binding assay results were normalized to the initial fluorescence reading. All experiments were repeated six times. K_observed_ (K_obs_) was obtained by fitting the data as a one phase-exponential decay using Graphpad Prism 7.

## Supplementary Material

Supplementary information
