# Supplementary material for: The unusual flagellar-targeting mechanism and functions of the trypanosome ortholog of the ciliary GTPase Arl13b
Source: J Cell Sci. 2018 Sep 5;131(17):jcs219071. doi: 10.1242/jcs.219071 (PMC6140319; doi:10.1242/jcs.219071)
Supplement: Supplementary information [file joces-131-219071-s1.pdf]

Table S1 List of plasmids used in the study

Purpose	Plasmid	Selection marker	Insert description
Endogenous tagging	pMOTag4YH-TbArl13	Hygromycin	TbArl13-YFP-3HA
	pPOTv6 3BB2-mNG-3BB2	Hygromycin	TbArl13-3BB2-mNG-3BB2
	pPOTv6 3BB2-mNG-3BB2	Blasticidin	TbArl3A-3BB2-mNG-3BB2
	pPOTv7 TagRFPt	Hygromycin	TagRFPt-TbArl3C
Inducible overexpression	pLEW-FL-YFP (Tet)	Phleomycin	TbArl13
	pLEW-DD-YFP(Tet)	Phleomycin	TbArl13(1-186)
	pLEW-CD-YFP(Tet)	Phleomycin	TbArl13(181-270)
	pDEX-mCherry-DDiR- (Cmt)	Phleomycin	mCherry-DDiR
	pDEX-TbArl3A-Q70L-BB2 (Tet)	Phleomycin	TbArl3A(Q70L)-BB2
	pDEX-TbArl3B(Q71L)-BB2 (Tet)	Phleomycin	TbArl3B(Q71L)-BB2
	pDEX-TbArl3C(Q77L)-BB2 (Tet)	Phleomycin	TbArl3C(Q77L)-BB2
Inducible overexpression (for RNAi complementation assays)	pDEX-FLiR-YFP(Cmt)	Blasticidin	TbArl13iR (1-270)
	pDEX-DDiR-YFP(Cmt)	Blasticidin	TbArl13iR (1-186)
	pDEX-CDiR-YFP(Cmt)	Blasticidin	TbArl13iR (181-270)
	pDEX-CCtiR-YFP(Cmt)	Blasticidin	TbArl13iR (1-244)
	pDEX-R133QiR-YFP(Cmt)	Blasticidin	TbArl13(R133Q)iR
	pDEX-Tb24-CDiR-YFP(Cmt)	Blasticidin	Tb24-CDiR (187-270)
BioID	pDEX-FLiR-MycBirA (Cmt)	Blasticidin	TbArl13iR
BiFC	pLEW-FLiR-VN(Tet)	Phleomycin	TbArl13iR
	pDEX-TbArl3A-VC (Cmt)	Blasticidin	TbArl3A
	pDEX-TbArl3B-VC (Cmt)	Blasticidin	TbArl3B
	pDEX-TbArl3C-VC (Cmt)	Blasticidin	TbArl3C
Overexpression	pXS2-TbArl3A-YFP	Blasticidin	TbArl3A
	pXS2-TbArl3B-YFP	Blasticidin	TbArl3B
	pXS2-TbArl3C-YFP	Blasticidin	TbArl3C
	pHD1034-YFP-RSP3	Puromycin	RSP3
RNAi	p2T7-TbArl13(Tet)	Phleomycin	TbArl13 CDS (nucleotides 160-593)
Bacterial expression	pET-28b-TbArl13	Kanamycin	His-TbArl13
	pGEX-6P-1-TbArl3A	Ampicillin	GST-TbArl3A
	pGEX-6P-1-TbArl3C	Ampicillin	GST-TbArl3C

Journal of Cell Science • Supplementary information

Table S2 List of antibodies used in the study

Antibodies	Antigen	Labelled Organelle/ structure	Origin/ Clonality	Reference/ Company	Used for
L3B2	FAZ1	FAZ filament	Mouse/ monoclonal	(Kohl et al., 1999)	IF (1:25)
YL1/2	Tyrosinated α-tubulin	basal body	Rat/ monoclonal	Santa Cruz (#sc-53029)	IF (1:2000)
anti-PAR	PAR	PFR	Mouse/ monoclonal	(Ismach et al., 1989)	IF (1:500)
anti-PFR1	PFR1	PFR	Rat/ polyclonal	Unpublished	IB (1:5000) IF (1:10000)
anti-TbBiP	BiP	ER	Rabbit/ polyclonal	(Bangs et al., 1993)	IB (1:1000)
anti-α-tubulin	alpha-tubulin	microtubule	Mouse/ monoclonal	B-5-1-2/Santa Cruz (#sc-23948)	IB (1:10000)
anti-TbArl13	TbArl13	axoneme	Rabbit/ polyclonal	This study	IF (1:500) IB (1:2000)
anti-Calflagin Tb24	Calflagin Tb17, Tb24, Tb44	-	Mouse /monoclonal	(Tyler et al., 2009)	IB (1:2000)
anti-YFP	YFP	-	Rabbit/ polyclonal	Unpublished	IB (1:1000)
anti-GFP	GFP	-	Mouse/ monoclonal	Roche #11814460001	IF (1:500)
anti-His	His tag	-	Mouse/ monoclonal	GE Healthcare (#27-4710-01)	IB (1:10000)
anti-HA	HA tag		Mouse/ monoclonal	Santa Cruz (#sc-7392)	IF (1:500)
anti-BB2	BB2 tag (also known as Ty1 tag)		Mouse/ monoclonal	(Bastin et al., 1996)	IB (1:500)
anti-mCherry	mCherry		Rabbit/polyclonal	Thermo Fisher (#PA5-34974)	IB (1:10000)

IF: immunofluorescence; IB: immunoblotting

Journal of Cell Science • Supplementary information

A

Full length

IDENTITY STATS:

min = 17.83
max = 65.65
mean = 31.7206666666667
stddev = 16.8911007995992

**TbArl13**	100%					
**HsArl13b**	26.29%	100%				
**DrArl13b**	27.4%	64.37%	100%			
**XlArl13b**	26.66%	65.65%	64.61%	100%		
**CeArl-13**	20%	20.54%	18.37%	20.27%	100%	
**CrArl13b**	24.81%	26.4%	28.25%	24.36%	17.83%	100%
	**TbArl13**	**HsArl13b**	**DrArl13b**	**XlArl13b**	**CeArl-13**	**CrArl13b**

SIMILARITY STATS

min = 27.83
max = 100
mean = 51.57
stddev = 26.692538699461

**TbArl13**	100%					
**HsArl13b**	38.51%	100%				
**DrArl13b**	37.77%	74.2%	100%			
**XlArl13b**	38.14%	74.76%	76.41%	100%		
**CeArl-13**	29.25%	28.1%	27.83%	29.45%	100%	
**CrArl13b**	36.66%	35.28%	39.06%	34.74%	28.1%	100%
	**TbArl13**	**HsArl13b**	**DrArl13b**	**XlArl13b**	**CeArl-13**	**CrArl13b**

GTPase/Arl domain

IDENTITY STATS:

min = 25.13
max = 82.68
mean = 41.7493333333333
stddev = 20.3735192399895

**TbArl13**	100%					
**HsArl13b**	34.1%	100%				
**DrArl13b**	32.94%	78.21%	100%			
**XlArl13b**	34.1%	82.12%	82.68%	100%		
**CeArl-13**	26.58%	26.25%	25.13%	26.25%	100%	
**CrArl13b**	29.65%	41.27%	40.69%	40.69%	25.58%	100%
	**TbArl13**	**HsArl13b**	**DrArl13b**	**XlArl13b**	**CeArl-13**	**CrArl13b**

SIMILARITY STATS

min = 36.41
max = 100
mean = 61.8016666666667
stddev = 23.8846552902346

**TbArl13**	100%					
**HsArl13b**	49.13%	100%				
**DrArl13b**	47.39%	86.03%	100%			
**XlArl13b**	47.39%	89.38%	89.38%	100%		
**CeArl-13**	36.41%	38.54%	38.54%	39.1%	100%	
**CrArl13b**	43.02%	55.23%	56.97%	56.97%	38.95%	100%
	**TbArl13**	**HsArl13b**	**DrArl13b**	**XlArl13b**	**CeArl-13**	**CrArl13b**

B

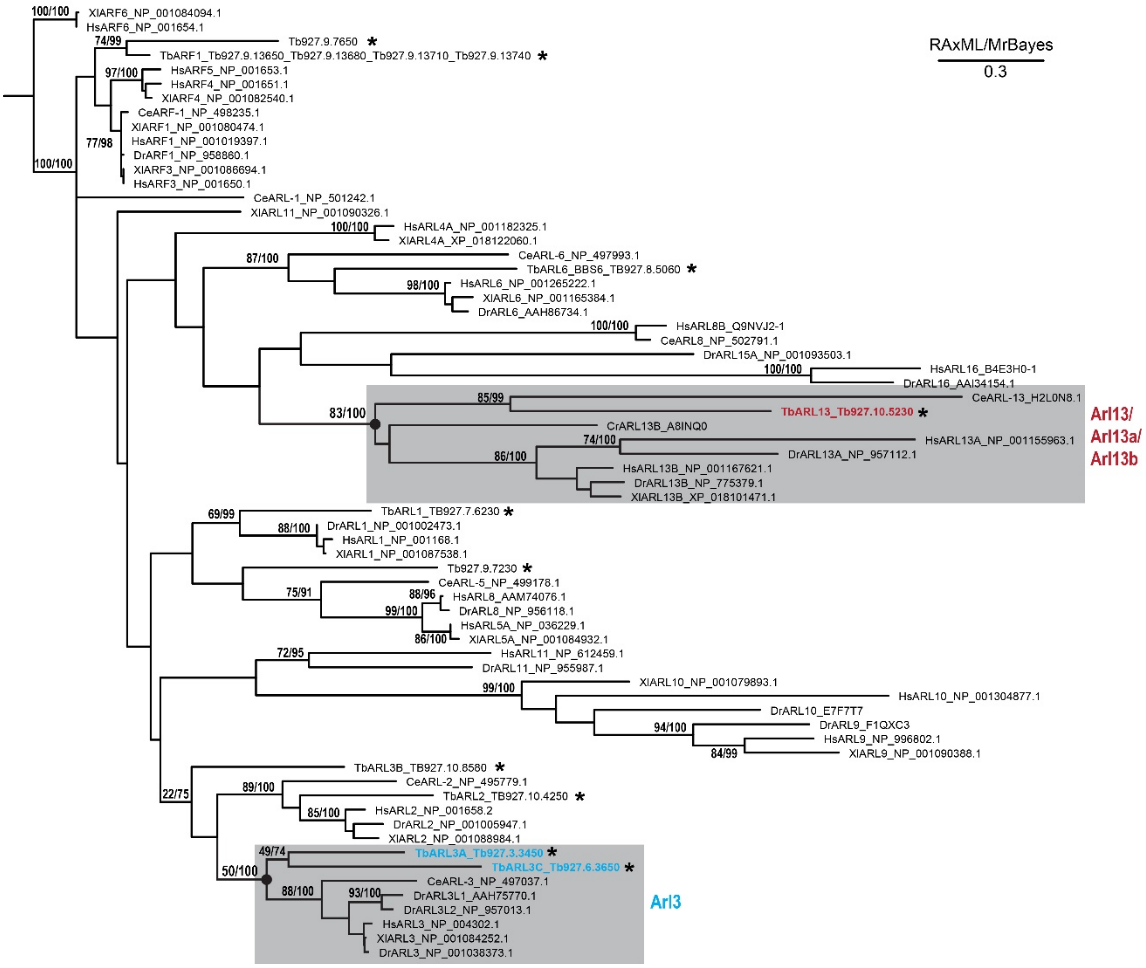

Fig. S1 TbArl13 is the Arl13b orthologue in *T. brucei*. (A) Cross comparison of identity and similarity between TbArl13 and other previously published Arl13b proteins. Both full-length amino acid sequences (left) and GTPase domain sequences (right) were

Journal of Cell Science • Supplementary information

analyzed. (B) Amino acid maximum likelihood topology of all predicted Arf/Arl proteins in the *T. brucei* genome and representative Arf/Arl family members from other eukaryotic groups. TbArl13 (red) robustly forms a clade with other Arl13/Arl13a/Arl13b proteins. TbArl3A and TbArl3C (blue) also fall under the Arl3 clade (blue). ML bootstrap support values and Bayesian posterior probabilities are mapped if >=70 across both analyses. Support values for other relevant nodes are included. Major Arf/Arl members from human (H. *sapiens,* Hs), zebrafish (D. *rerio*, Dr), amphibians (X. *laevis,*Xl) and nematodes(C. *elegans*, Ce) and green algae (C. *reinhardtii,* Cr) were used for the reconstruction. Asterisks indicate *T. brucei* proteins. Branch support values calculated using Maximum-Likelihood (RAxML) and Bayesian Interefence (MrBayes) are shown.

Journal of Cell Science • Supplementary information

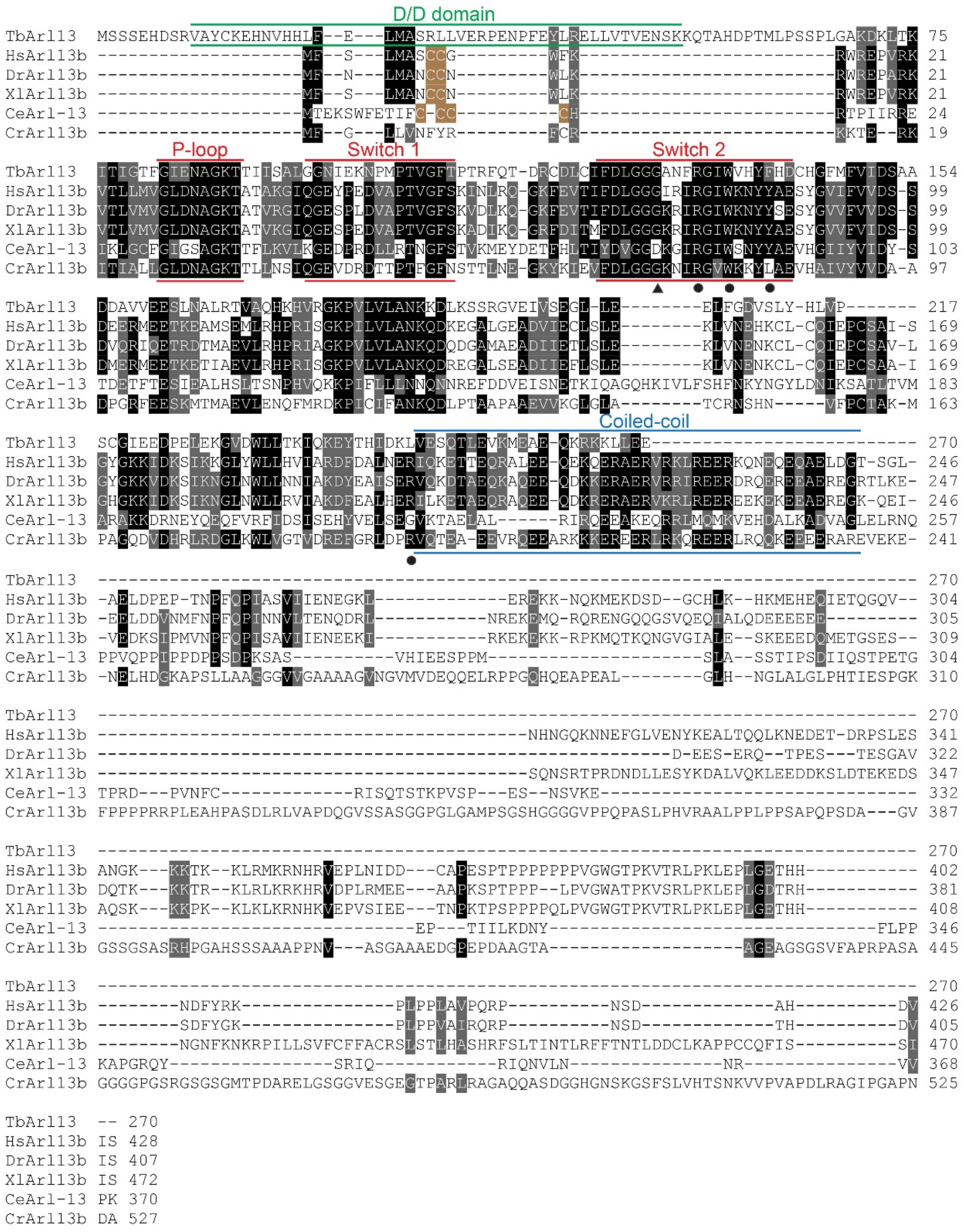

Fig. S2 Multiple sequence alignment of TbArl13 and Arl13b proteins from other organisms. TbArl13 protein sequence is aligned with Arl13b from H. *sapiens* (Hs), D. *rerio* (Dr), X. *laevis, (*Xl), C. *elegans* (Ce) and C. *reinhardtii* (Cr). Black triangle indicates the missing catalytic glutamine (GXXGQ) in all Arl13b proteins. Black dots indicate residues that have been found mutated in JS patients. Highlight in brown are the

Journal of Cell Science • Supplementary information

cysteine palmitoylation sites conserved in Ar113bs, but not found in CrArl13 and TbArl13. Residues conserved/similar across at least 4 out of 6 sequences are shaded in black/grey respectively.

Journal of Cell Science • Supplementary information

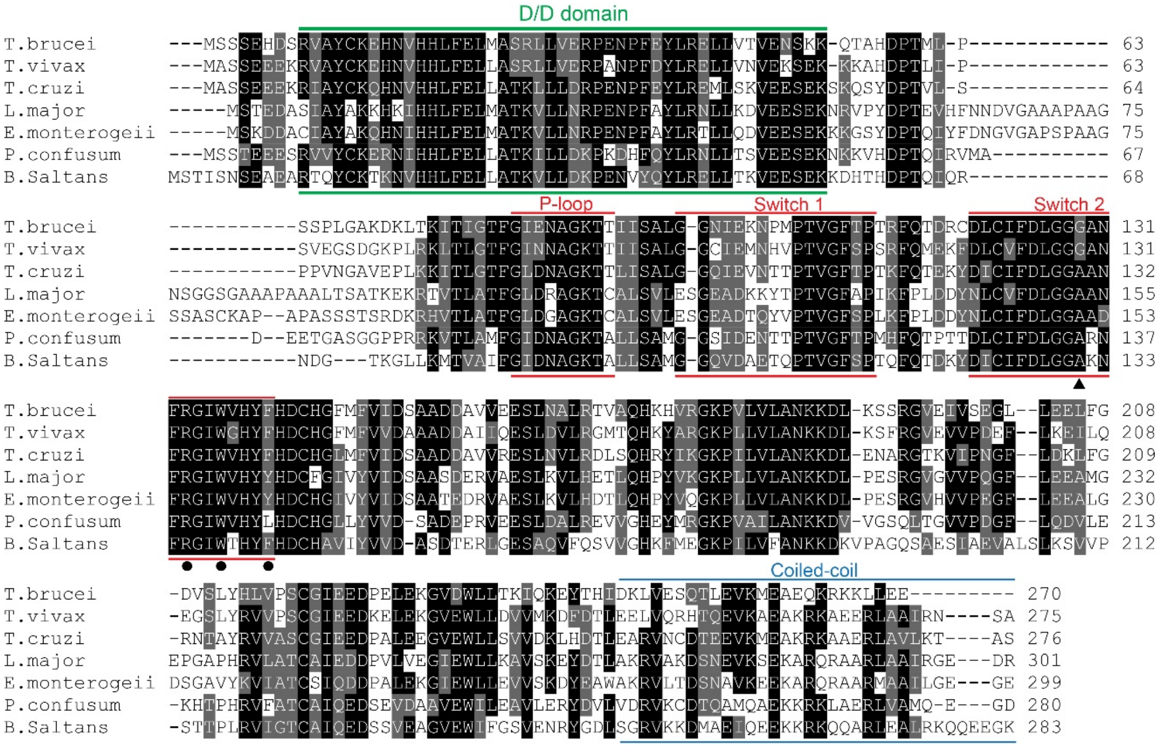

Fig. S3 The N-terminal D/D domain is conserved in kinetoplastid Arl13 proteins. Multi-sequence alignment of Arl13 from five different kinetoplastid species. Black arrowhead indicates the missing catalytic glutamine (GXXGQ) in all known Arl13b orthologues. Black circles indicate residues that have been found mutated in JS patients. Residues conserved/similar across at least 5 out of 7 sequences are shaded in black/grey respectively.

Journal of Cell Science • Supplementary information

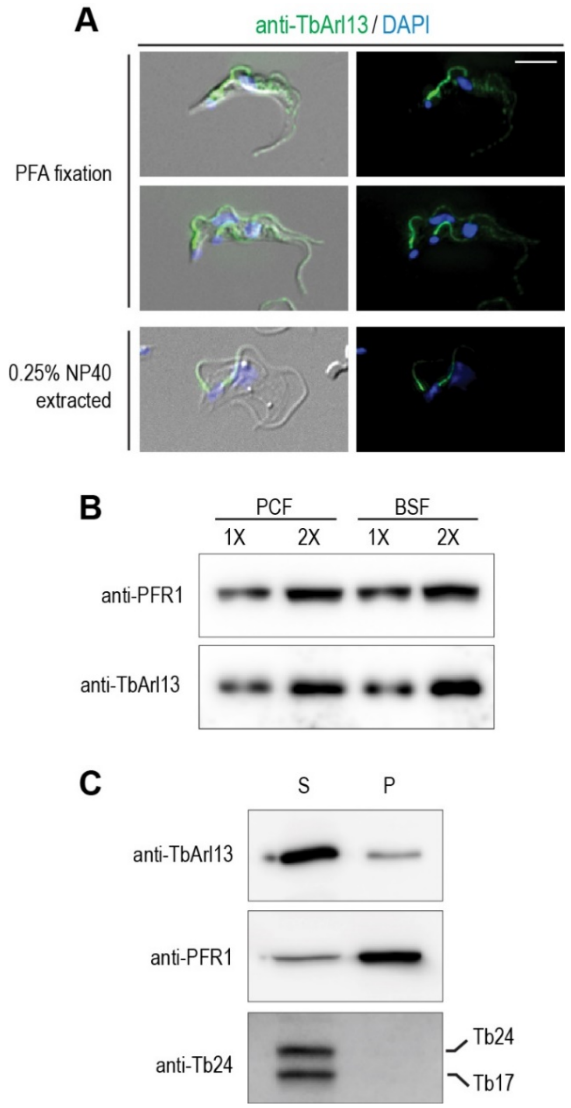

Fig. S4 TbArl13 is expressed and localizes to the flagellum in the BSF form. (A) BSF cells were fixed with 4% PFA either before (top) or after extraction with 0.25% NP40 (bottom) and stained with anti-TbArl13 (green) and DAPI (blue). Scale bar = 5µm. (B) The expression level of TbArl13 in BSF is comparable to that in PCF. '2X' indicates twice the amount of cell lysate loaded in the well compared to '1X'. Anti-PFR1 was used as a loading control. (C) BSF cells were extracted using 1% Triton X-100 in PBS under 37°C to maximize removal of flagellar membrane associated proteins. Calflagin Tb24 and its paralogue Tb17 is solely found in the S (supernatant) fraction, while a portion of TbArl13 is consistently found in the P (pellet) fraction. PFR1 was used as a marker for the flagellar cytoskeleton.

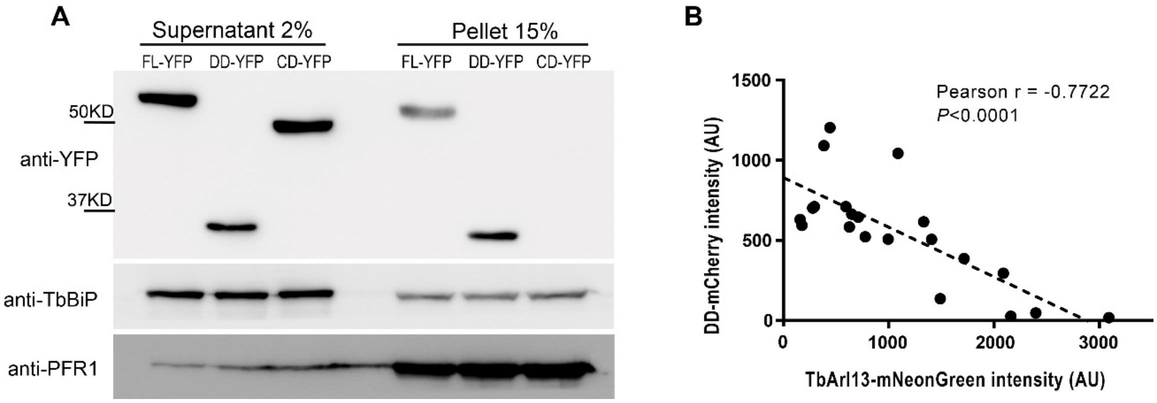

Fig. S5 D/D mediated flagellar targeting. (A) Fractionation analysis of FL-YFP, DD-YFP and CD-YFP proteins. Both FL-YFP and DD-YFP but not CD-YFP associates with the cytoskeleton pool. For each cell type, $2x10^8$ cells were extracted with 1% TritonX-100 in PBS before centrifugation to yield the supernatant and pellet fractions. Anti-TbBiP and anti-PFR1 were used as loading and extraction controls. (B) In relation to Fig. 2(E). In cells co-expressing TbArl13-mNeonGreen and mCherry-DD, the level of mNeonGreen measured on the axoneme is negatively correlated to the level of mCherry. Pair number n= 21, correlation coefficient = -0.7722. $P<0.0001$.

Journal of Cell Science • Supplementary information

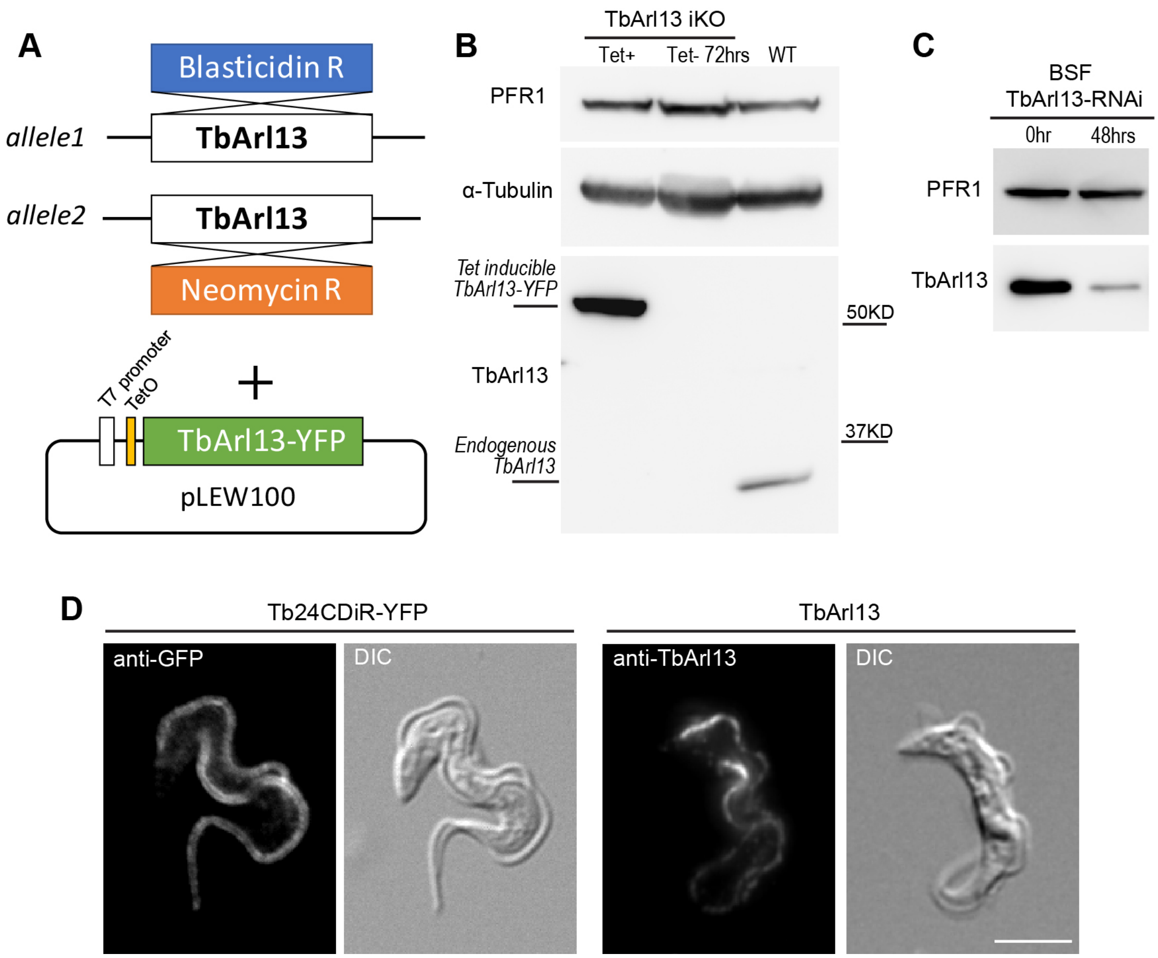

Fig. S6 TbArl13 iKO, RNAi and Tb24CDiR-YFP localization. (A) Schematic diagram showing the construction of TbArl13-iKO cells. Tetracycline (Tet)-inducible TbArl13b-YFP was stably introduced into cells with both endogenous TbArl13 alleles replaced with blasticidin and phleomycin resistant genes, respectively. (B) Immunoblots confirm the absence of endogenous TbArl13 in iKO cells compared to wild type (WT) cells. 72 hours after the removal of tetracycline, TbArl13-YFP expression could not be detected. (C) Immunoblots confirm the reduction of TbArl13 in BSF cells, 48hrs following the induction of TbArl13-RNAi. For all immunoblots, anti-TbArl13 was used to detect both endogenous TbArl13b and recombinant TbArl13-YFP. Anti-PFR1 and anti-alpha-tubulin were used as loading controls. (D) Comparison of the immunofluorescence-labeling of the flagellar membrane localizing chimera mutant Tb24CDiR and WT TbArl13. Tb24CDiR-YFP-expressing and wild type (WT) BSF cells were fixed and immune-stained with anti-GFP and anti-TbArl13, respectively. Tb24CDiR-YFP could be seen outlining the flagella, a pattern that is typical of flagellar membrane association but is not observed with WT TbArl13. Scale bar=5µm.

Journal of Cell Science • Supplementary information

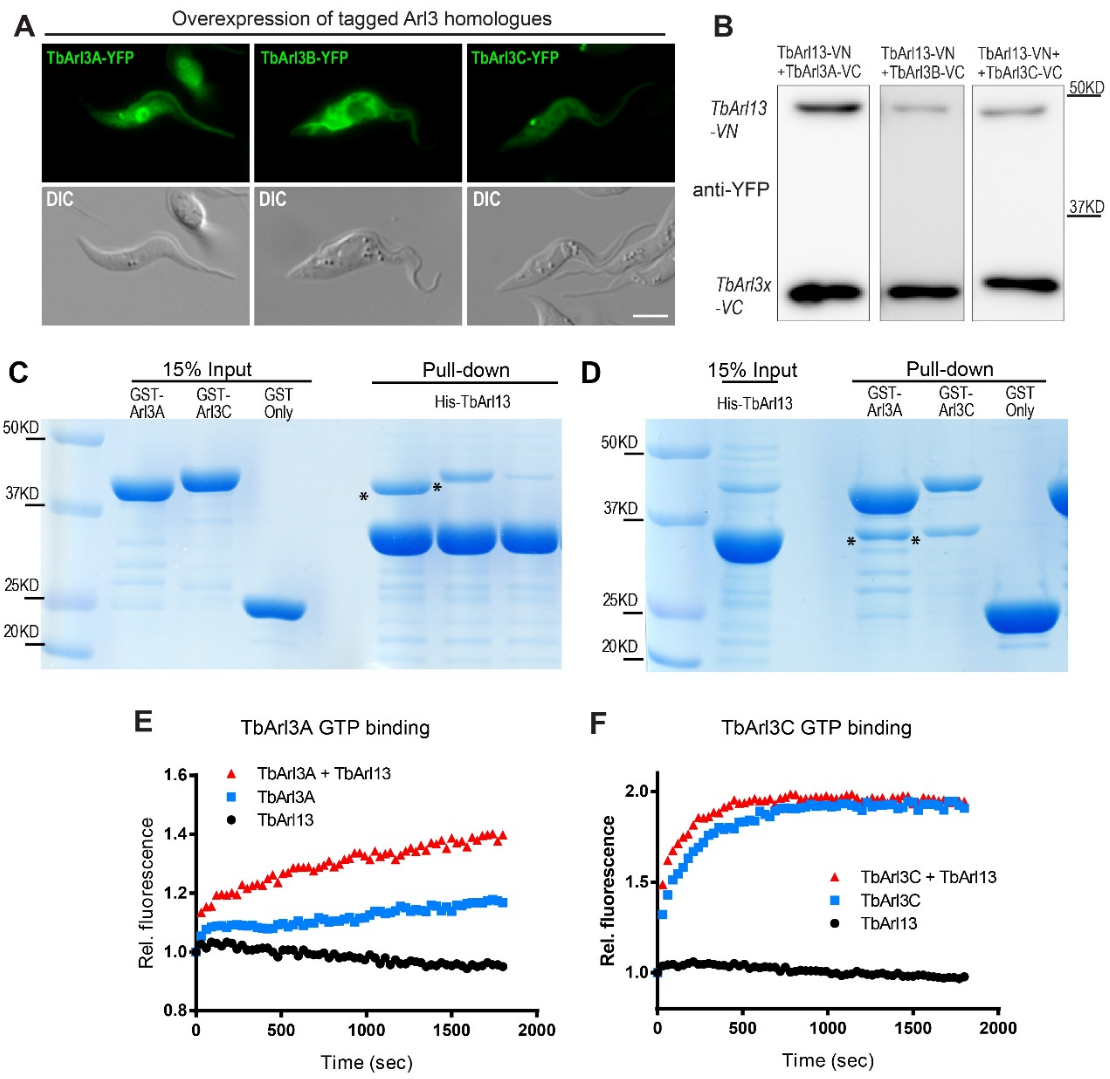

Fig. S7 GTP binding of TbArl3A and TbArl3C is accelerated by TbArl13. (A) TbArl3A-YFP, TbArl3B-YFP and TbArl3C-YFP are expressed in PCF cells. DNA is labeled using DAPI. Scale bar = 5μm. (B) In relation to Fig. 5(B), the expression of TbArl13-VN, TbArl3A-VC, TbArl3B-VC and TbArl3C-VC were confirmed by immunoblots. Both VN and VC fragments could be recognized by anti-YFP polyclonal antibodies. (C, D) Affinity purified His-TbArl13 can pull-down affinity purified GST-TbArl3A and GST-TbArl3C (C), and vice versa (D). * marks the pull-down band. (E, F) Addition of His-TbArl13 promotes mant-GTP binding to TbArl3A and TbArl3C. Consistent with Fig. 5(E), TbArl3C has an exceptionally high intrinsic GTP binding rate compared to TbArl3A.

Journal of Cell Science • Supplementary information

References

Bangs, J. D., Uyetake, L., Brickman, M. J., Balber, A. E. and Boothroyd, J. C. (1993). Molecular cloning and cellular localization of a BiP homologue in Trypanosoma brucei. Divergent ER retention signals in a lower eukaryote. *J Cell Sci* **105 (Pt 4)**, 1101-13.

Bastin, P., Bagherzadeh, A., Matthews, K. R. and Gull, K. (1996). A novel epitope tag system to study protein targeting and organelle biogenesis in Trypanosoma brucei. *Molecular and biochemical parasitology* **77**, 235-239.

Ismach, R., Cianci, C. M., Caulfield, J. P., Langer, P. J., Hein, A. and McMahon-Pratt, D. (1989). Flagellar membrane and paraxial rod proteins of Leishmania: characterization employing monoclonal antibodies. *J Protozool* **36**, 617-24.

Kohl, L., Sherwin, T. and Gull, K. (1999). Assembly of the paraflagellar rod and the flagellum attachment zone complex during the Trypanosoma brucei cell cycle. *The Journal of Eukaryotic Microbiology* **46**, 105-109.

Tyler, K. M., Fridberg, A., Toriello, K. M., Olson, C. L., Cieslak, J. a., Hazlett, T. L. and Engman, D. M. (2009). Flagellar membrane localization via association with lipid rafts. *Journal of cell science* **122**, 859-866.
